# Intravenous iron isomaltoside versus oral iron supplementation for treatment of iron deficiency in pregnancy: protocol for a randomised, comparative, open-label trial

**DOI:** 10.1186/s13063-020-04637-z

**Published:** 2020-08-26

**Authors:** Veronika Markova, Rebecka Hansen, Lars Lykke Thomsen, Anja Pinborg, Torben Moos, Charlotte Holm

**Affiliations:** 1 Department of Obstetrics and Gynaecology, Amager-Hvidovre Hospital, Copenhagen University Hospital, Kettegaard Allé 30, 2650 Hvidovre, Denmark; 2 Pharmacosmos A/S, Roervangsvej 30, 4300 Holbaek, Denmark; 3 Laboratory of Neurobiology, Department of Health Science and Technology, Aalborg University, Fredrik Bajers Vej 7, 9220 Aalborg, Denmark; 4 Department of Health sciences, University of Copenhagen, Blegdamsvej 3B, 2200 Copenhagen, Denmark; 5 Fertility Clinic, Juliane Marie Centre, Rigshospitalet, University of Copenhagen, Blegdamsvej 9, 2100 Copenhagen, Denmark

**Keywords:** Iron deficiency, Iron deficiency anaemia, Pregnancy, Ferric derisomaltose, Iron isomaltoside 1000, Randomised controlled trial

## Abstract

**Background:**

Iron deficiency is common in pregnancy. If left untreated, iron deficiency can lead to iron deficiency anaemia, which is a condition related to maternal and neonatal morbidity. The prevalence of iron deficiency increases through the trimesters, which means that women with iron deficiency in the beginning of pregnancy also have a great risk of developing iron deficiency anaemia during pregnancy. Standard treatment is oral iron in individualised intensified doses based on screening values in 1st trimester.

Maternal symptoms of iron deficiency and iron deficiency anaemia include fatigue, reduced physical performance, and restless legs syndrome (RLS). Severe anaemia may cause dizziness, dyspnea, palpitation, orthostatism, and syncope, and it decreases the woman’s ability to cope with blood loss during delivery. The anaemia may also compromise contractility in the uterine musculature increasing the risk for prolonged labour, caesarean section, and postpartum haemorrhage. Foetal iron deficiency may cause low birthweight and adversely affect foetal and early childhood brain development with long-term deficits.

**Methods:**

In this randomised comparative, open-label, single-centre, phase IV trial, 200 pregnant women between 14 and 21 weeks of gestation who have iron deficiency after 4 weeks of standard treatment will be randomised 1:1 to either a single 1000 mg dose of intravenously administered ferric derisomaltose/iron isomaltoside 1000 or a fixed dose of 100 mg oral ferrous fumarate containing 60 mg ascorbic acid.

The primary endpoint is to prevent iron deficiency anaemia defined by a low level of haemoglobin throughout the trial. Other endpoints include other haematological indices of iron deficiency and anaemia, clinical outcomes by questionnaires, and collection of adverse events. Explorative endpoints by medical record follow-up include complications up to 7 days after delivery.

**Discussion:**

This trial will provide evidence on how to prevent iron deficiency anaemia. The trial population represents a clinical reality where pregnant women often have sustained iron deficiency despite an increased oral iron dose. Thus, this evidence can be used to consider the optimal 2nd line of treatment in iron-deficient pregnant women.

**Trial registration:**

European Union Drug Regulating Authorities Clinical Trials Database 2017-000776-29. Registered on 3 May 2017.

ClinicalTrials.gov NCT03188445. Registered on 15 June 2017.

## Administrative information

Note: the numbers in curly brackets in this protocol refer to SPIRIT checklist item numbers. The order of the items has been modified to group similar items (see http://www.equator-network.org/reporting-guidelines/spirit-2013-statement-defining-standard-protocol-items-for-clinical-trials/).

**Table:** 

Title (1)	Intravenous iron isomaltoside versus oral iron supplementation for treatment of iron deficiency in pregnancy: protocol for a randomised, comparative, open-label trial
Trial registration {2a and 2b}.	European Union Drug Regulating Authorities Clinical Trials Database: 2017-000776-29 ClinicalTrials.gov: NCT03188445.
Protocol version {3}	Trial ID: P-Monofer-PREG-01 version 6, amendment 5, date of protocol: 4 September 2019 (accepted by the Scientific Ethics Committees on 13 November 2019)
Funding {4}	The trial is fully funded and sponsored by Pharmacosmos A/S, Roervangsvej 30, 4300 Holbaek, Denmark. The trial participants are not compensated financially for participating in the trail.
Author details {5a}	^1^ Department of Obstetrics and Gynaecology, Amager-Hvidovre Hospital, Copenhagen University Hospital, Kettegaard Allé 30, 2650 Hvidovre, Denmark ^2^ Pharmacosmos A/S, Roervangsvej 30, 4300 Holbaek, Denmark ^3^ Laboratory of Neurobiology, Department of Health Science and Technology, Aalborg University, Fredrik Bajers Vej 7, 9220 Aalborg, Denmark ^4^ University of Copenhagen, Department of Health sciences, Blegdamsvej 3B, 2200 Copenhagen, Denmark ^5^ Fertility Clinic, Juliane Marie Centre, Rigshospitalet, University of Copenhagen, Blegdamsvej 9, 2100, Copenhagen, Denmark
Name and contact information for the trial sponsor {5b}	Pharmacosmos A/S, Roervangsvej 30, 4300 Holbaek, Denmark. Tel.: +45 59 48 59 59
Role of sponsor {5c}	Pharmacosmos A/S was the GCP-sponsor and funded the study. Pharmacosmos A/S took responsibility for and coordinated data collection and management and will take responsibility for and coordinate analysis and interpretation of data and writing of the clinical study report. Pharmacosmos A/S was involved in preparation of this manuscript. Pharmacosmos cannot prohibit the trial center or investigator from publishing trial data.

## Introduction

### Background and rationale {6a}

Untreated iron deficiency (ID) in pregnancy can lead to iron deficiency anaemia (IDA) [1, 2]. ID in pregnancy is defined by low iron stores, measured by a level of circulating ferritin < 30 μg/L [3–6]. Iron is essential for basic cellular processes, such as cell division and the synthesis of haemoglobin (Hb) [7]. Anaemia in pregnancy is defined by a Hb level < 11.0 g/dL, WHO [1, 5]. Thus, IDA in pregnancy is defined as Hb < 11.0 g/dL and serum-(*s*)-ferritin < 30 g/μg/L.

In Danish pregnant women, who do not take iron supplementation, approximately 50% have ID and 21% have IDA [8]. Anaemia (regardless the cause) is estimated to occur in 24% of Danish pregnant women (WHO).

The prevalence of ID is approximately 7% in 1st trimester, 14–40% in 2nd trimester, and 30–62% in 3rd trimester [9, 10]. Prevalence of IDA is approximately 2% in 1st trimester, 8% in 2nd trimester and, 27% in 3rd trimester [11, 12]. The steep increase in ID and IDA through the trimesters illustrates that women with ID in the beginning of pregnancy have a great risk of developing IDA during pregnancy.

National recommendations on routine iron supplementation in pregnancy do not necessarily prevent ID and IDA as seen in a Swizz study, where approximately 32% of pregnant women in 2nd trimester had ID and 6.2% IDA despite a recommended daily oral iron dose of 80 mg [13].

Maternal symptoms of ID and IDA include fatigue/exhaustion, reduced physical performance, restless legs syndrome (RLS), and decreased ability to cope with blood loss during delivery. Severe anaemia may cause dizziness, shortness of breath, palpitation, orthostatism, and syncope [14, 15]. Iron is an essential part of myoglobin, which explains why ID may compromise contractility in the uterine musculature that in turn may increase the risk for prolonged labour, caesarean section, and postpartum haemorrhage [16, 17]. The consequence of maternal anaemia for the foetus is low birthweight, which can complicate the neonatal period [18–21]. ID during foetal life may adversely affect brain development with long-term deficits. One trial showed that maternal anaemia in pregnancy could be linked to 14% of cases of mental retardation at a 7-year follow-up [22].

Thus, isolated ID can be symptomatic and adversely affect foetal brain development. ID is a precursor for IDA, which is associated with maternal and foetal/neonatal morbidity. If treated early in pregnancy, the consequences of ID can be prevented.

In clinical practice, the standard treatment of ID and IDA in pregnancy recommended by the Danish Society of Obstetricians and Gynaecologists (DSOG) is oral iron supplementation in individualised doses based on screening values of Hb and ferritin in 1st trimester [23].

Oral iron supplementation is associated with gastrointestinal adverse effects, affecting patient compliance [14]. Thus, not all women treated with oral iron will respond to or comply with treatment. The recommendations are not clear on how to act in case of failure of the standard treatment. Thus, a pregnant woman with sustained ID will most likely be recommended to remain on oral iron and only receive intravenous (IV) iron if she becomes anaemic. However, it has not yet been investigated what treatment—intensified oral iron or IV iron—is most favourable in case the recommended standard treatment is not sufficient.

Treatment with intravenous ferric derisomaltose/iron isomaltoside 1000 (FDI) (Monofer®/Monoferric®, Pharmacosmos, Holbaek) is indicated when oral treatment is ineffective and when rapid iron delivery is clinically indicated. FDI is one of the newer IV iron formulations available. It was initially launched in Europe in 2010 and consists of iron and a carbohydrate moiety where the iron is tightly bound in a matrix structure. It is the matrix structure that enables a controlled and slow release of iron to iron-binding proteins, avoiding potential toxicity from release of labile iron [24]. FDI has been studied in non-clinical reprotoxicology trials. In supra therapeutic doses, foetal malformations were seen in rabbits [Monofer® Investigators Brochure]. The risk for teratogenic or foeto-toxic effects is considered minimal at the proposed therapeutic dose. Several studies of FDI treatment of iron deficiency anaemia have been published without detected unexpected safety issues [25–31]. FDI should be confined to second and third trimester if the benefit is judged to outweigh the potential risk for both the mother and the foetus [6, 32, 33].

Ferrous fumarate containing ascorbic acid denotes a combination product administered in a film-coated tablet for oral ingestion. Ferrous fumarate is a ferrous salt. Ascorbic acid (vitamin C) facilitates iron uptake by maintaining iron in its ferrous form [34, 35].

Previous trials have investigated the use of different IV iron preparations in pregnant women with IDA [36–42]. We have not been able to identify trials investigating the use of IV iron for prevention of IDA in iron-deficient pregnant women. A recent trial where pregnant women with IDA were treated with the IV iron formulation ferric carboxymaltose reported a statistically significant improvement in Hb change from baseline in the IV iron group compared to oral iron group at 6 weeks. Overall hypersensitivity reactions were rare, IV iron treatment had a beneficial effect on Hb increase, and neonatal outcomes did not differ between groups [40].

There are several reasons why it is important to systematically investigate if the use of IV iron is an effective and attractive mode of treatment in pregnancy compared to intensified oral iron treatment in women with sustained ID despite standard treatment: ID rarely resolves despite standard treatment, is unlikely to resolve later in pregnancy if still present after 4 weeks of standard care, is often left untreated in cases of intolerance to oral iron, is unfavourable for the foetus, and can progress into severe ID and IDA in later trimesters.

Therefore, measures to effectively treat ID early to prevent IDA are of great clinical importance.

### Objectives {7}

In order to prevent IDA throughout the trial in pregnant women, who have ID after 4 weeks of standard treatment, the primary objective is to compare the efficacy of a single dose of IV administered FDI with a fixed dose of oral ferrous fumarate containing ascorbic acid.

The secondary objectives are to compare the effect of IV FDI and oral ferrous fumarate with ascorbic acid on (i) haematological indices of IDA in maternal blood, (ii) maternal fatigue and quality of life (QoL), (iii) RLS, (iv) the need for an additional IV FDI dose or (rescue) red blood cell (RBC) transfusion, and (v) safety.

### Trial design {8}

The trial is a randomised, comparative, open-label, single-centre, phase IV trial, with planned 200 women randomised 1:1 to a single dose of IV FDI or a fixed dose of oral iron ferrous fumarate with ascorbic acid. The randomisation is stratified by Hb value (≥ or < 11.0 g/dL).

## Methods: participants, interventions, and outcomes

### Study setting {9}

The trial is being conducted at the Department of Obstetrics and Gynecology at Copenhagen University Hospital, Amager-Hvidovre Hospital, which is a tertiary hospital with 7000–8000 yearly deliveries.

Healthy women who wish to participate and fulfil the inclusion criteria are enrolled at gestational age (GA) 14 + 0–21 + 0. The duration for each participant is approximately 18 weeks and involves four follow-up visits by a trial investigator at the Amager-Hvidovre Hospital.

### Eligibility criteria {10}

Pregnant women at GA 14 + 0–21 + 0 are eligible for inclusion if they fulfil the following criteria: *s-*ferritin < 30 μg/L (0–29 μg/L) after 4 weeks of standard treatment, age ≥ 18 years, and signed informed consent form.

The exclusion criteria are as follows: multiple pregnancies; history of anaemia not caused by iron deficiency; iron overload or disturbances in utilisation of iron; previous hypersensitivity to IV iron or to any excipients in the investigational drug products; active asthma within the last 5 years; multiple allergies; known decompensated liver cirrhosis or active hepatitis; active acute or chronic infections (assessed by clinical judgement); rheumatoid arthritis with symptoms or signs of active inflammation; treated with IV iron products, blood transfusion, or erythropoietin within 4 weeks prior to inclusion; participation in any other interventional trial where the trial drug has not passed 5 half-lives prior to inclusion; any other medical condition that, in the opinion of the investigator, may cause the participant to be unsuitable for the completion of the trial or place the participant at potential risk from being in the trial; meeting RBC transfusion criteria (Hb ≤ 6.9 g/dL = ≤ 4.3 mmol/L with intolerable symptoms of anaemia or an Hb ≤ 6.4 g/dL (≤ 4.0 mmol/L) regardless of anaemia symptoms; and inability to read and understand the Danish language.

### Who will take informed consent? {26a}

Trial investigators will obtain informed consent.

### Additional consent provisions for collection and use of participant data and biological specimens {26b}

On the consent form, the participants will be informed about the use and storage of personal data collected during their participation in the trial, i.e. until completion of trial or withdrawal of consent, this in accordance with the General Data Protection Regulation (GDPR). The consent form contains information concerning the personnel who can access personal data collected during this trial, i.e. investigators, monitor, and regulatory authorities.

By signing the informed consent form, the participants agree to the terms addressed in the form.

This trial involves collecting biological specimens for storage.

## Interventions

### Explanation for the choice of comparators {6b}

The choice of the comparator is based on current standard treatment of ID in pregnancy.

### Intervention description {11a}

A. IV FDI administered at the baseline visit as a single dose of 1000 mg (if pre-pregnancy body weight < 50 kg then 20 mg/kg pre-pregnancy body weight). The dose is diluted in 100 ml 0.9% sodium chloride and is given over approximately 20 min.
B. Fixed dose oral ferrous fumarate (100 mg elemental iron) with ascorbic acid 60 mg [34], once daily.

### Criteria for discontinuing or modifying allocated interventions {11b}

If the participant has IDA at *T*_6w_ or *T*_12w_ defined as Hb < 11 g/dL and *s-*ferritin < 30 μg/L, an additional dose of IV iron isomaltoside is allowed in both groups (not at *T*_18w_ due to lack of AE monitoring). The maximum cumulative dose is set to 2000 mg for the individual participant.

### Strategies to improve adherence to interventions {11c}

Compliance to oral treatment is encouraged at every follow-up visit.

### Relevant concomitant care permitted or prohibited during the trial {11d}

Iron supplementation other than study treatment is prohibited.

### Provisions for post-trial care {30}

There is no anticipated harm and compensation for trial participation. Post-trial, the pregnant women will be cared for in a clinical setting, i.e. standard antenatal care, where both intervention and comparator therapies are available.

### Outcomes {12}

The primary endpoint of this trial is a Hb ≥11.0 g/dL (≥ 6.8 mmol/L) at all trial visits post-baseline (*T*_3w_, *T*_6w_, *T*_12w_, and *T*_18w)._

The secondary endpoints are Hb ≥ 11.0 g/dL (≥ 6.8 mmol/L) at each follow-up visit, change in Hb and other haematological indices of ID/IDA from baseline to each follow-up visit (e.g. reticulocytes, reticulocyte haemoglobin content (CHr), *s*-ferritin, *s*-transferrin, *s*-iron, hepcidin, and calculated transferrin saturation (TSAT)), incidence of hypophosphatemia (defined as serum [*s*]*-*phosphate < 2 mg/dL) from baseline to each follow-up visit, change in fatigue and quality of life questionnaires from baseline to each follow-up visit, presence of RLS at each follow-up visit, number of participants who receive an additional IV iron isomaltoside dose at *T*_6w_ and/or *T*_12w_, reason for the additional IV iron isomaltoside dose (non-compliance, lack of effect), number of participants receiving RBC transfusions and the number of RBC units per transfused participant from baseline to final subject visit, type and incidence of AEs observed at any time until final subject visit, serious or severe hypersensitivity reaction starting at or after the first dose of randomised treatment, AEs of special interest (constipation, diarrhoea, flatulence, nausea, vomiting, abdominal pain, dyspepsia, dysgeusia, and stool discoloration), number of participants who discontinue from the trial because of lack of response or intolerance of investigational drugs, foetal bradycardia related to infusion of IV iron isomaltoside at or after GA 26, change in biochemical safety parameters from baseline to each follow-up visit, and compliance to treatment: at baseline in the IV group and at *T*_6w_ and *T*_18w_ in the oral group.

Specific explorative endpoints registered between final subject visit up until 7 days postpartum will be extracted from medical records: incidence of maternal antepartum haemorrhage, thromboembolic events, gestational diabetes mellitus, gestational hypertensive disorders, blood transfusions, prolonged labour, length of labour, oxytocin use, assisted delivery, unplanned caesarean section, postpartum haemorrhage, maternal blood loss at labour (ml), length of maternal and neonatal hospital admission, maternal death, incidence of intrauterine growth retardation, preterm birth, ante- or postnatal asphyxia, low birthweight, neonatal infection, neonatal anaemia, required paediatric assistance, admission to neonatal ward, congenital malformations, foetal or neonatal death, GA at delivery, date of delivery and birthweight.

### Clinical outcome measures

The FACIT-fatigue questionnaire has been validated in a mixed population of patients with IDA. The questionnaire consists of 13 items regarding fatigue. Each item has a response scale of 0–4, 0 being ‘not at all’ and 4 being ‘very much so’. The score for each item is added up to a summed score, where a low score equals more fatigue and higher score equals a better condition. Selected items are there for reversed when adding up the total score.

The Short Form 12 (SF-12) is used to evaluate quality of life (QoL). The SF-12 is derived from the more comprehensive SF-36. Both SF-12 and SF-36 cover the two summary measures of physical and mental health through eight domains/scales (physical functioning, physical role, bodily pain, general health, vitality, social function, emotional role, mental health) which in turn are covered by 12 items. Higher scores indicate better conditions.

RLS is evaluated by using the four diagnostic criteria [43] as a quantitative outcome which is either present or not present.

### Participant timeline {13}

**Fig. 1:**
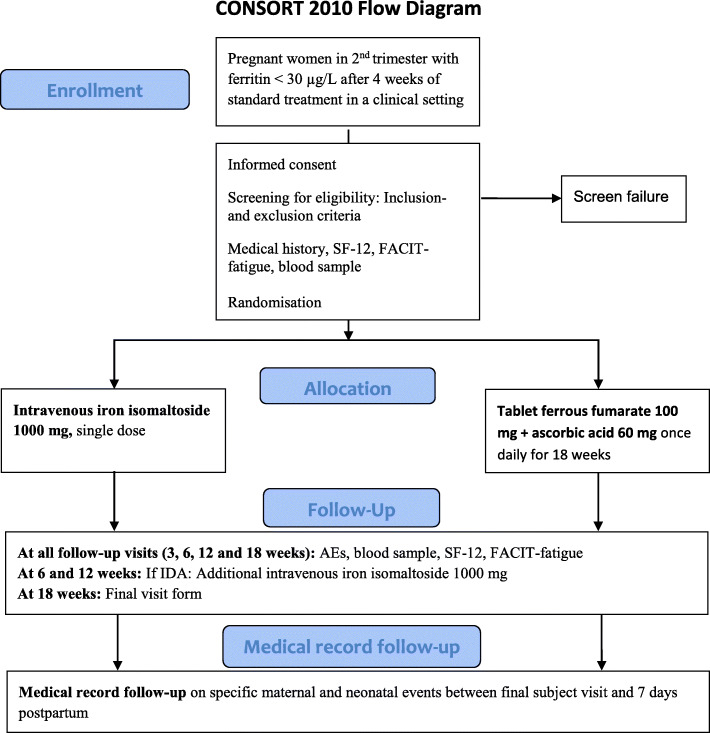
Illustration of trial flow (SF-12, Short Form 12; FACIT-fatigue, questionnaire on fatigue; AEs, adverse events; IDA, iron deficiency anaemia)

**Fig. 2:**
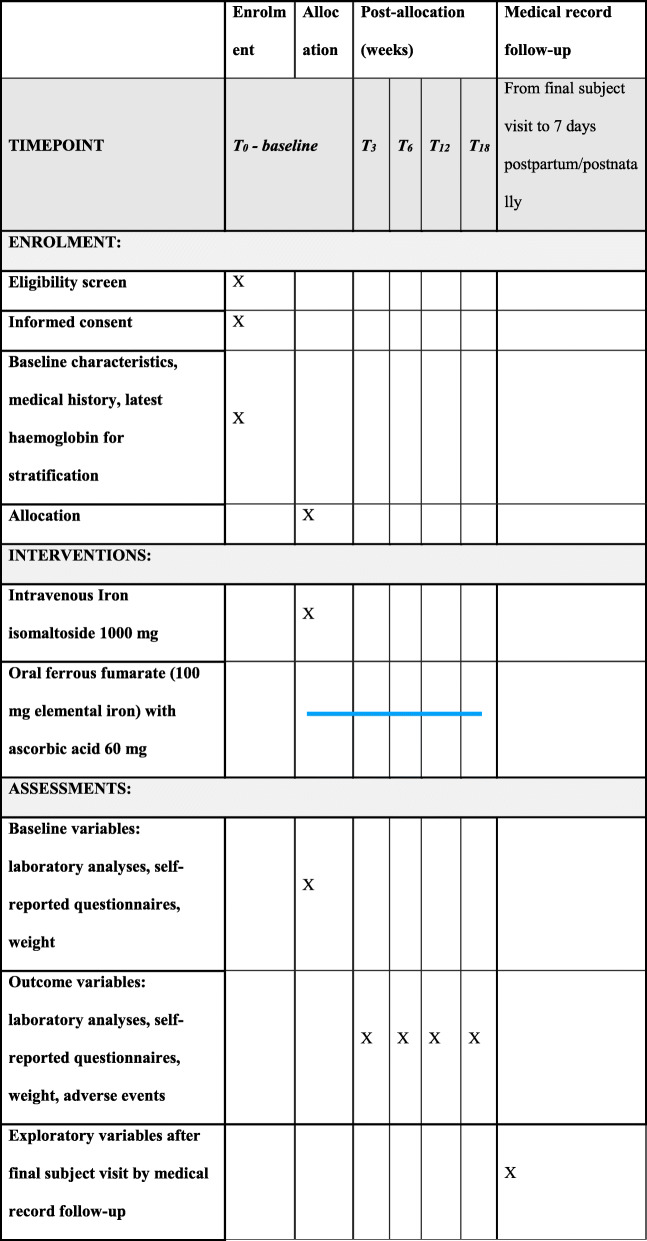
Schedule for enrolment, intervention, and assessment during the trial

The participants will attend the baseline visit and 4 follow-up visits: *T*_3w_ 3 weeks (± 2 days), *T*_6w_ 6 weeks (± 3 days), *T*_12w_ 12 weeks (± 1 week), and *T*_18w_ 18 weeks (± 1 week) from baseline (see Figs. 1 and 2).

### Sample size {14}

The sample size calculations are based on the primary endpoint: Hb ≥ 11 g/dL (≥ 6.8 mmol/L) at all time points after baseline [1, 5]. The primary endpoint will be estimated by a Kaplan-Meier curve. Participants who drop out during the trial will be accounted for (set as ‘censored’) by the methodology and therefore not addressed specifically in the sample size calculations. It is assumed that 5% and 17.5% of the participants in the FDI and oral treatment groups respectively will have a Hb < 11 g/dL at some point during the trial, corresponding to 95% and 82.5% will have a Hb ≥ 11 g/dL at all post-baseline visits. Using a significance level of 5%, and setting the power to 80%, 100 participants in each treatment group are required in order to detect the assumed difference between FDI and oral iron.

### Recruitment {15}

The women will be recruited from the clinical setting. Standard clinical procedures for pregnant women at the trial site include 1st trimester screening for iron deficiency and anaemia by measuring Hb and serum ferritin. Women with ID or IDA are recommended intensified oral iron supplementation and repeated blood samples in the 2nd trimester. Women with persistent iron deficiency despite this standard oral treatment are contacted, informed about the trial, and are invited to the baseline visit.

## Assignment of interventions: allocation

### Sequence generation {16a}

The procedure for preparing the randomisation list is approved by the Global Trial Responsible Statistician. The randomisation list is prepared by BioStata ApS, Birkeroed, Denmark.

### Concealment mechanism {16b}

The allocation list is imputed in the electronic Case Report File (eCRF) and lies prepared within the eCRF. The sequence list is not accessible to the investigators.

### Implementation {16c}

The investigators consecutively invited all eligible participants to attend the baseline visit. At the baseline visit, demographics and latest Hb level are entered in the eCRF prior to randomisation. Thereafter, the participant is randomised stratified by Hb value and proceeds to the allocated treatment.

## Assignment of interventions: blinding

### Who will be blinded {17a}

This trial is open label. Neither the outcome assessors nor data analysts are blinded.

### Procedure for unblinding if needed {17b}

The design is open label so unblinding will not occur.

## Data collection and management

### Plans for assessment and collection of outcomes {18a}

Data is collected during the five trial visits and by medical record follow-up. Source data is defined as all information in original records or certified copies of original records of clinical findings, observations, or other activities in a clinical trial necessary for reconstruction and evaluation of the trial. Source data is entered from paper source into an eCRF.

Laboratory analysis data is drawn electronically from the hospitals IT system to the eCRF.

### Plans to promote participant retention and complete follow-up {18b}

The participant has the right to withdraw from the trial at any time and for any reason without prejudice to her future medical care by the physician or at the institution. If a participant withdraws from the trial, the investigator will perform all final visit assessments besides the scheduled trial assessments for that visit.

As part of the subject information, the purpose of the trial and the importance of completing the trial will be explained to the subjects. Subject compliance (oral iron group) will be followed throughout the trial and advices and guidelines provided in case of non-compliance. Both treatment groups will be in regular contact with the investigator.

In extraordinary/force major situations, the principal investigator can temporarily suspend trial visits, e.g. if entering the hospital carries a risk for the participant. In such case, we will attempt to collect information via telephone or email, where possible, and schedule trial-related laboratory analyses simultaneously with any pre-scheduled appointments in a clinical setting. The authorities will be informed promptly in case of such temporary restructuring actions, including the reason for the decision.

### Data management {19}

Data management is outsourced. The data collection tool for this trial is the eCRF, which is compliant with 21 CFR Part 11 regulations. Clinical data management is performed in accordance with applicable standards and data cleaning procedures.

### Confidentiality {27}

Data are handled confidentially and stored in a locked archive.

### Plans for collection, laboratory evaluation, and storage of biological specimens for genetic or molecular analysis in this trial/future use {33}

Blood samples will be collected by venous puncture at every participant visit. Haematological indices of ID/IDA as well as safety indices will be analysed within hours and evaluated by the investigators during the next working day at the latest. A single tube containing plasma from each visit will be stored at the trial site and will be used to assess hepcidin at an external laboratory. The samples will be destroyed after analysis or latest at completion of the trial. Thus, specimens collected at trial visits will not be used for genetic analyses or analyses in any future trials.

## Statistical methods

### Statistical methods for primary and secondary outcomes {20a}

Time to Hb < 11 g/dL and proportion of participants with correction of anaemia (Hb ≥ 11.0 g/dL) at *T*_3w_, *T*_6w_, and *T*_12w_ will be estimated by a Kaplan-Meier curve using scheduled visits. Based on the Kaplan-Meier curve, the proportion of participants who have met the primary endpoint (achievement/maintenance of Hb ≥ 11 g/dL at all post-baseline visits) at *T*_18w_ will be estimated and compared between the treatment groups.

The primary endpoint analysis will be performed for the intention-to-treat analysis set and will be repeated for the full analysis set and per protocol analysis sets.

As sensitivity, the risk difference will be used to compare the proportion of participants with Hb ≥ 11 g/dL at all post-baseline visits. Risk difference and the associated 95% Newcombe CI and *p* value will be calculated, adjusting for strata (Hb </≥ 11 g/dL at inclusion) using the Cochran-Mantel-Haenszel method. In this analysis, participants who do not complete the study period will be set as failures.

The change in Hb and biochemical safety parameters from baseline to *T*_3w_, *T*_6w_, *T*_12w_, and *T*_18w_ will be analysed using a restricted maximum likelihood-based mixed model for repeated measures approach.

The proportion of participants who (a) receive an additional IV FDI dose, (b) receive allogenic RBC transfusions, (c) discontinue from the trial because of lack of response or intolerance of investigational drugs, and (d) develop hypophosphatemia will be compared between the treatment groups by Fisher’s exact test.

The number of units of RBC transfused per transfused participant from baseline to final subject visit will be compared between the treatment groups by an analysis of variance or Wilcoxon test.

For participants in the oral iron group, AEs with onset at/after the time of first additional IV iron isomaltoside dose will be excluded from the main AE summaries. In addition, displays of all AEs per overall treatment group will be produced, as well as summaries and participant listings of AEs occurring at/after the time of first additional IV iron isomaltoside dose.

Medical record follow-up will be tabulated by descriptive statistics.

### Interim analyses {21b}

No interim analyses are planned. The trial sponsor and the principal investigator can temporarily suspend or prematurely discontinue the trial at any time for reasons such as safety, ethical issues, severe non-compliance, and insufficient subject enrolment. The authorities will be informed promptly in case of suspension or termination, including the reason for the decision.

### Methods for additional analyses (e.g. subgroup analyses) {20b}

No additional analyses are planned.

### Methods in analysis to handle protocol non-adherence and any statistical methods to handle missing data {20c}

The intention-to-treat (ITT) analysis set will include all subjects as randomised. The per protocol (PP) analysis set will include all subjects who do not have any major protocol or GCP deviation of clinical or statistical significance. Non-compliance, i.e. treatment dose outside the 80–120% range within the first 6 weeks of treatment, is considered a major protocol deviation.

All participants in the intention-to-treat analysis set with post-baseline Hb data will be included with their observed data. Participants without post-baseline Hb values will have change from baseline set to 0 at the first post-baseline visit. The model will include the fixed, categorical effects of treatment, week, and treatment-by-week interaction as well as the continuous, fixed covariates of baseline Hb value and baseline Hb-by-week interaction. Similar analyses will be performed for the other haematological indices (reticulocytes etc.), change in fatigue, QoL, and RLS.

### Plans to give access to the full protocol, participant level-data, and statistical code {31c}

The protocol, individual subject data, and statistical codes will not be available. The primary and secondary endpoints will be uploaded to EudraCT in accordance with national requirements.

## Oversight and monitoring

### Composition of the coordinating centre and trial steering committee {5d}

Pharmacosmos sponsors the trial and the responsibility for the quality and integrity of the trial data resides with Pharmacosmos. Pharmacosmos has a Quality Management System in place to ensure that the trial is conducted, and data are generated, documented (recorded), and reported in compliance with the protocol, International Conference on Harmonization-Good Clinical Practice (ICH-GCP), and the applicable regulatory requirements. The oversight of data quality will be provided by a trial core team who consist of personnel responsible for project management, medical monitoring, statistics, data management, medical writing, GCP quality control, and GCP quality assurance. The responsibility of the trial core team is to ensure high data quality, regulatory compliance, scientific validity, and transparency in all activities in the clinical trial via robust planning and timely action. The trial core team will meet regularly to follow-up on the trial progress. Monitoring, data management, and statistics is handled by Vendors, but the ultimate responsibility for the quality and integrity of the trial data always resides with Pharmacosmos.

All trial-specific monitoring information and procedures are described and controlled via a monitoring guideline and a project plan. All monitoring reports are reviewed and approved by the Pharmacosmos project director. A risk management plan ensures ongoing risk assessment and adequate mitigation throughout the trial.

The investigator is responsible for trial execution at site including enrolment of trial subjects and collecting informed consent. Subjects receive both written and oral information about the trial, and they are giving time for consideration and questions before providing written consent. The consent procedure for each subject is checked as part of the Source Data Review/Source Data Verification as part of the monitoring procedure.

### Composition of the data monitoring committee, its role and reporting structure {21a}

There will not be selected a data monitoring committee. Instead, the oversight of data quality will be provided by a trial core team. Roles and responsibilities of the trial core team are described in section 5d—the “Composition of the coordinating centre and trial steering committee” section.

### Adverse event reporting and harms {22}

AEs are collected and evaluated for relatedness to trial drug, seriousness, severity, expectedness, and outcome. AE are defined in the ICH-GCP guideline.

### Frequency and plans for auditing trial conduct {23}

To ensure compliance with ICH-GCP guidelines and all applicable regulatory requirements, Pharmacosmos A/S, its designee, or its regulatory agencies may conduct a regulatory inspection of this trial, in which case direct access to all source data and documents is mandatory.

### Plans for communicating important protocol amendments to relevant parties (e.g. trial participants, ethical committees) {25}

Protocol amendments will be submitted to the Ethical Committee and Danish Medicines Agency and implemented after approval.

### Dissemination plans {31a}

The results of the trial, positive as well as negative, will be published by the end of the trial. Also, the findings of this trial will be presented as part of a PhD thesis and defence.

The primary endpoint and key secondary endpoints (AEs and change in Hb, serum ferritin, and TSAT from baseline to *T*_3w_, *T*_6w_, *T*_12w_, and *T*_18w_) will be uploaded on public websites according to national requirements.

## Discussion

The objective of this study is to compare IV FDI to oral iron therapy for the prevention of IDA in iron-deficient pregnant women.

We use the first-choice treatment method as a comparator in our trial. Therefore, we will be able to use the study results for assessing the potential benefit of IV FDI in a clinical setting.

In the first approved version of our protocol, we aimed to recruit women with IDA. However, we soon discovered that IDA is rare in 1st and beginning of 2nd trimester in Danish women. Thus, to recruit pregnant women with IDA, we either would have to examine women in 3rd trimester or prepare for a very long enrolment period (i.e. a decade). At this point, it became clear that a preventive approach would be beneficial for both the pregnant women and for the progression of our trial. This resulted in a trial design aiming to prevent IDA, as opposed to treating IDA after manifestation. It is well known that IDA causes morbidity [14–22]. However, previous studies have focused on treatment [36–42]. We have focused on prevention, thereby minimising the damage that IDA can cause before effectively treated. From a clinical perspective, it makes good sense to avoid the development of morbidity rather than treating it, once occurred. IV iron treatment is more expensive than oral treatment, both because of the cost of the actual medicine, but also costs associated with administration. However, IV iron might correct ID faster and have higher compliance than oral treatment and thereby reduce complications at delivery (i.e. length of hospital admission, surgery, RBC transfusions), making this a highly relevant trial in a socioeconomic context as well.

This trial stands out from other trials regarding the rather strict policy on the gestational age time window for the five trial visits, which we consider a great strength in our study design. We thereby acknowledge that pregnancy is a state with major haemodynamic, hormonal, and physiological changes. We know that haemoglobin drops in 2nd/3rd trimester as part of a physiological process which prepares the pregnant woman for the blood loss associated with delivery [44]. Also, pregnancy symptoms such as fatigue and nausea are more common in 1st trimester and tend to subside in 2nd trimester [45, 46]. By recruiting pregnant women who are all at approximately the same GA at inclusion and thereby also at the follow-up visits, we ensure that the participants are at the same physiological stages at the respective visits. We hypothesise that this will make both the laboratory and clinical result more homogeneous between the treatment groups in that all data is equally affected by the physiological deviations during a pregnancy.

We consider the evaluation of clinically relevant outcomes as a strength in our trial, as these outcomes are very important to investigate. Optimally, we would have used a tool that has been validated in this specific population (European iron-deficient/anaemic pregnant women), which could detect differences in symptoms of ID/IDA while accounting for pregnancy. However, such a tool that fulfils all these criteria does not exist. A recent review illustrates how sparse clinical outcome reporting generally is in trials on perinatal IDA [47].

We chose two self-administered questionnaires in the official Danish versions: FACIT-fatigue and SF-12 in their unedited form (copyright protected). Permission to use the questionnaires has been obtained from the official websites. Both questionnaires are validated in a broader population. This is a limitation in our trial.

We chose the FACIT-fatigue questionnaire because fatigue is considered the most common symptom of anaemia. The questionnaire contains questions that seem appropriate for a pregnant woman and is simple, short, and manageable.

For evaluation of QoL, we chose the 12-question version, rather than the 36-question version, because we did not want to risk the combined body of questionnaire material to cause drop out due to an overwhelming amount of questions at each visit. The SF-12 questionnaire addresses emotional wellbeing as part of quality of life assessment. This aspect of ID/IDA is of increasing interest, e.g. a recent study in women after postpartum haemorrhage showed that postpartum depression scores (Edinburgh Postnatal Depression Scale) improved significantly in the intravenous iron group compared to the standard treatment group [30].

The evaluation of RLS is based on four diagnostic questions, rather than using an available rather comprehensive questionnaire, which was a deliberate choice on our part to keep the combined body of questionnaire material to a manageable level.

As a habitually low Hb and a mild/moderate ID is often asymptomatic, the differences in clinical outcomes of ID/IDA can be difficult to detect. We therefore chose Hb for the primary outcome in this trial.

## Trial status

The first protocol version (P-Monofer-PREG-01 version 1) was approved on 7 April 2017.

The first participant was randomised on 11 December 2017. The final participant was randomised on 28 February 2020, thus approximately 2 months after the initial submission of this manuscript.

## Supplementary information

**Additional file 1.** . SPIRIT 2013 Checklist: Recommended items to address in a clinical trial protocol and related documents*

## Abbreviations

AE: Adverse event
CHr: Reticulocyte haemoglobin content
DMT1: Divalent metal transporter 1
DSOG: Danish Society of Obstetricians and Gynecologists
eCRF: Electronic case report file
FDI: Ferric derisomaltose/iron isomaltoside 1000
GA: Gestational age
Hb: Haemoglobin
ICH-GCP: International Conference on Harmonization-Good Clinical Practice
ID: Iron deficiency
IDA: Iron deficiency anaemia
IIM: Intravenous iron isomaltoside
IV: Intravenous
QoL: Quality of life
RBC: Red blood cell
RLS: Restless legs syndrome
SF: Short form
TSAT: Transferrin saturation
WHO: World Health Organization
